# *In situ* formation of artificial moth-eye structure by spontaneous nano-phase separation

**DOI:** 10.1038/s41598-018-19414-x

**Published:** 2018-01-18

**Authors:** Tong Li, Junhui He, Yue Zhang, Lin Yao, Tingting Ren, Binbin Jin

**Affiliations:** 1 Functional Nanomaterials Laboratory, Center for Micro/Nanomaterials and Technology, and Key Laboratory of Photochemical Conversion and Optoelectronic Materials, Technical Institute of Physics and Chemistry, Chinese Academy of Sciences, Zhongguancundonglu 29, Haidianqu, Beijing, 100190 China; 2 University of Chinese Academy of Sciences, Beijing, 100049 China

## Abstract

Unprecedented *in situ* formation of artificial moth-eye structure is demonstrated by spontaneous nano-phase separation of a silica-based system on substrate. The moth-eye thin film with a homogenously distributed nipples array shows broadband antireflection functionalities. The mechanism of nano-phase separation is unveiled as spinodal decomposition by chemical freezing method and thermodynamic analysis. The current method may provide a new avenue to ready fabrication of patterned nanostructures toward a variety of applications.

## Introduction

Phase separation is a physical phenomenon occurring in a thermodynamically unstable system and un-mixing of a homogeneous system to form new phases. The mechanisms of phase separation are classified into nucleation growth (NG) process and spinodal decomposition (SD) process based on the thermodynamic process^1,2^. In the phase diagram of a binary system, the nucleation growth takes place between binodal and spinodal lines, while spinodal decomposition takes place within the spinodal line^1^. Notably, SD produces a three-dimensional continual microstructure, making the material showing synergistic effects and good mechanical properties^2,3^.

Some morphologies have been obtained by the SD process^1,4–7^. The con-continuous films derived from SD of polymer blends show dispersive ordered/disordered structures and impressive interfacial and optical properties^7,8^ or synergistic effects^9,10^. However, the polymer blends are restricted by their sensitivity to pH, the low solubility in solvent (e.g. ethanol, acetone and water) and weak robustness of the prepared polymer films^2,6,9^. Nakanishi’s group reported a polymer-based silicane system of controlled SD phenomenon. It provides an approach to preparing dispersive macrostructural silica materials that demonstrate application prospects for adsorbents, membranes, filters and separation media^2,11,12^. In order to understand the evolution of domain microstructures (e.g. circles matrix, worm-like structure) observed during spinodal decomposition and coarsening, the SD phenomenon was also simulated by using computational algebraic topology on the basis of the deterministic Cahn–Hilliard theory^11,13^. Generally speaking, the previous works require proper selection of polymer precursors and/or complex preparation methods as well as cumbersome process of removing one of the polymer phases. To date, the preparation of various films by SD is mainly based only on organic polymers blend systems, limiting the development of multi-component con-continuous microstructures with good optical and mechanical properties or synergistic effects.

The moth-eye structure, which was firstly investigated by Clapham and Hutley^14^, has a nano-array structure with a gradient refractive index and large surface area^13–16^. Unlike single–layer antireflective thin films with narrowband antireflective properties, moth-eye structured thin films show broadband and wide-angle antireflective properties, having wide application prospects in solar cells, optical lens and displays^16–18^.

To date, moth-eye structures have been obtained by complex/expensive template method^19–21^, reactive ion etching^22^ and lithography^16,23^, but have never, to our best knowledge, been prepared by the SD process.

Here, we developed a novel facile polymer-free approach to *in situ* formation of con-continuous microstructural surfaces from a hybrid silicane system by surface spontaneous nano-phase separation on various substrates. After washing and calcination, a con-continuous moth-eye structure was surprisingly obtained.

## Results and Discussion

**Figure 1:**
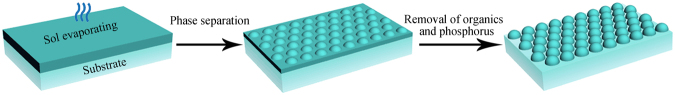
Schematic of *in situ* formation of artificial moth-eye structure by surface spontaneous nano-phase separation.

**Figure 2:**
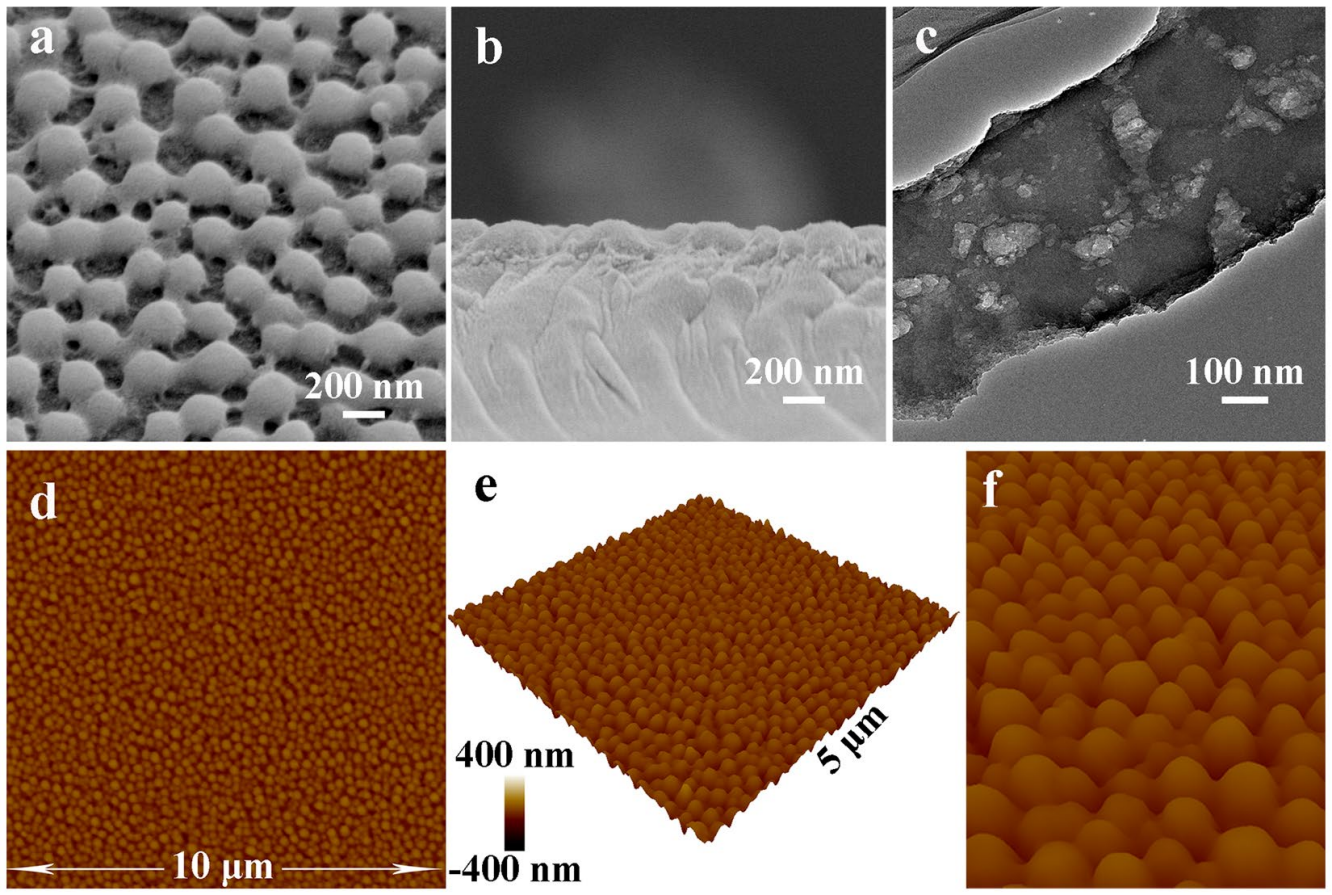
Morphology of the artificial moth-eye film. Tilted view (**a**) and cross-section (**b**) SEM and TEM (**c**) images of the film. Two- and three-dimensional and zoom-in (**d**,**e** and **f**) AFM images of the film. Red arrow and black arrow in (**c**) point to solid nipple and nanoporous gap, respectively.

**Figure 3:**
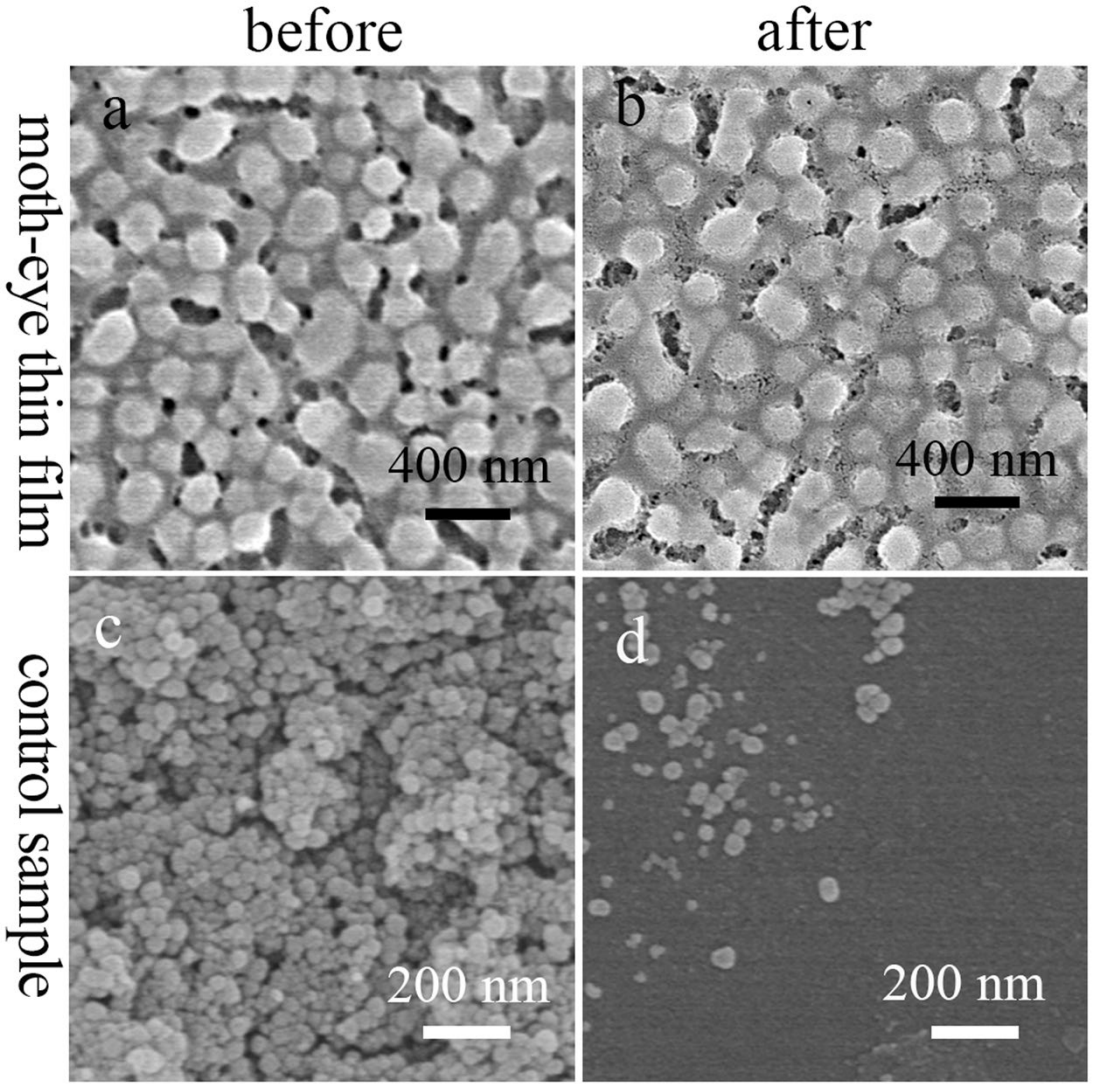
SEM images of coatings on glass substrates before (**a**–**c**) and after (**b**,**d**) sand abrasion test, respectively. (**a**,**b**) moth-eye thin film, (**c**,**d**) control sample prepared by coating silica nanoparticles on glass substrate.

The formation process of the moth-eye structure is shown in Fig. 1. The aged precursor sol, which consists of hybrid silica oligomers, alcohol, cetyltrimethylammonium bromide (CTAB), water and H_3_PO_4_, is deposited on substrate by dip-coating or spray-coating. Then a dispersive array spontaneously appears upon solvent evaporation at room temperature under a 20% relative humidity. Finally, an artificial moth-eye structure forms after removing phosphorus and CTAB by water cleaning and heat treatment. The morphology and structure of an optimal moth-eye film were investigated by scanning electron microscopy (SEM), transmission electron microscopy (TEM) and atomic force microscopy (AFM), and shown in Fig. 2. Tilted and cross-sectional SEM images (Fig. 2a,b) show that the film consists of a single-layer array of dispersive cross-linked hemispherical nipples. The diameter of nipples is ca. 182 nm, the distance between nipples is ca. 259 nm and the thickness of the film is ca. 170 nm. Figure 2c and Figure S1 further reveal that the film consists of solid nipples and nanoporous gaps between the nipples. The nanoporous gaps have a hierarchically porous structure with larger pores (7.4–20.0 nm) distributed in a nanoporous layer of ca. 1.2 nm pores. Two- and three-dimensional AFM images (Fig. 2d–f) as well as cross-sectional SEM image (Fig. 2b) reveal that the single-layer nipples are uniformly dispersed on substrate in the whole area. The height of nipples was estimated by AFM analysis (the height profile of a nipple is shown in Figure S2 as an example) and is ca. 146 nm, which is 24 nm less than the film thickness (170 nm) from the SEM image (Fig. 2b). The reason may be that the height of nipples by AFM is the distance from the top of nipples to the surface of nanoporous gaps, but the film thickness is a distance from substrate to the top of nipples. Hence, the artificial moth-eye film is composed of a nanoporous layer with dispersive nipples homogeneously arranged on it. Furthermore, the mechanical strength of the moth-eye thin film and the thin films consisting of accumulated silica nanoparticles was compared. Figure 3 show the SEM images of moth-eye thin film (Fig. 3a,b) and silica nanoparticles coatings (Fig. 3c,d) before and after sand abrasion test, respectively. In contrast to the common SiO_2_ nanoparticles coating heavily damaged (Fig. 3d) after the sand abrasion test and with weak strength^24,25^, the moth-eye thin film was hardly damaged after the test (Fig. 3b), indicating the moth-eye thin film has excellent resistance to sand abrasion. The moth-eye surface has a cross-linked structure, resulting in a higher mechanical strength.

**Figure 4:**
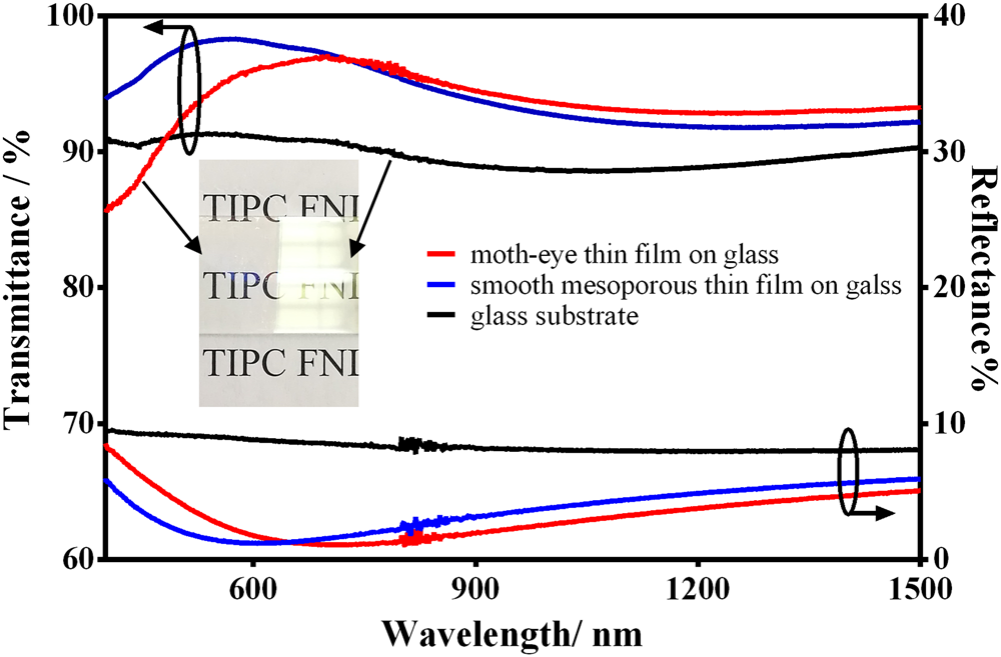
Transmission and reflection spectra of moth- eye thin film on slide glass (red line) in contrast to smooth mesoporous thin film on glass (blue line) and glass substrate (black line). The inset is a digital photo of moth-eye film coated glass in contrast to glass substrate under a daylight lamp.

The moth-eye thin films were dip-coated on two sides of glass substrates for the investigation of their optical properties. As shown in Fig. 4, the moth-eye film exhibits excellent broadband antireflective (AR) properties in comparison with bare substrate. In detail, the moth-eye film coated slide glass has the maximum transmittance of 97.2% (700 nm) and average transmittances of 94.1% (400–800 nm) and 93.7% (400–1500 nm) in contrast to slide glass with the maximum transmittance of 91.4% (533 nm) and average transmittances of 90.8% (400–800 nm) and 89.7% (400–1500 nm), while it has the minimum reflection of 1.1% (690 nm) and average reflectances of 2.8% (400–800 nm) and 3.3% (400–1500 nm) in contrast to slide glass with the minimum reflection of 7.7% (1257 nm) and average reflectances of 8.6% (400–800 nm) and 8.1% (400–1500 nm). Honestly, the low transmittance and high reflectance of the thin film in 400~600 nm are the disadvantages for the practical application in solar cells, which may due to the light scattering by the nipples and the large thickness of the thin film^26,27^. Furthermore, the refractive index of the moth-eye thin film was estimated by Equation 1^18^, and it is 1.319, which is a little higher than the refractive index (1.22) of optimal single-layer antireflective thin films on commercial glass^18,28^.

1 $${\rm{r}}={[\tfrac{{n}_{air}{n}_{s}-{n}^{2}}{{n}_{air}{n}_{s}+{n}^{2}}]}^{2}$$

**Figure 5:**
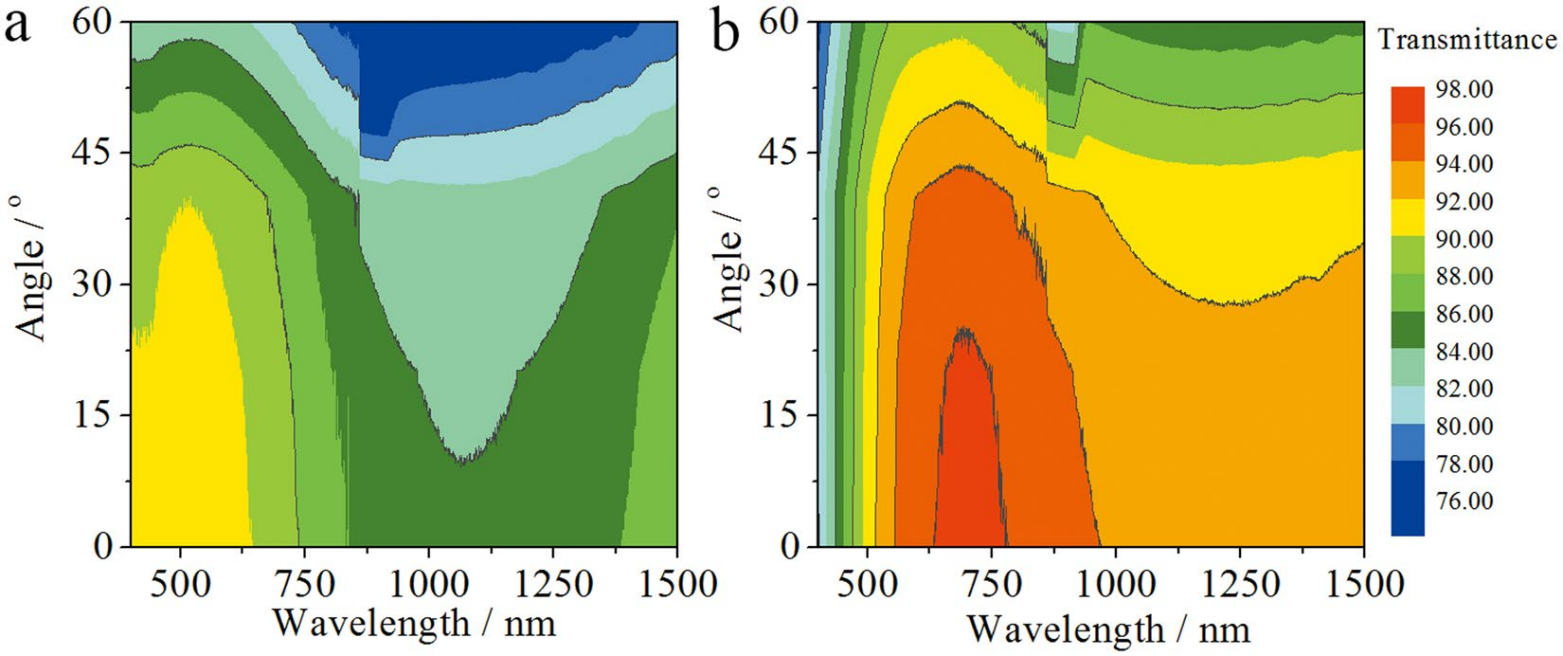
Contour plots of transmittance as a function of both wavelength and angle of incident light for (**a**) glass substrate and (**b**) moth-eye thin film on glass, respectively.

Where, r is the reflectance of one side of thin film, n_s_, n_air_ and n are the refractive index of the substrate, air and film, respectively. In fact, the moth-eye thin film has a gradient refractive index, which endows the coated glass with a low reflection (2.8%). Meanwhile, as shown in Fig. 4, the moth-eye thin film also shows a more broadband AR property in contrast to the smooth nanoporous AR film derived only from a proper porosity^29^ in the wavelength range of 750–1500 nm, indicating the nipples array of the film with a moth-eye effect also contributes to the optical properties besides the nanopores. In addition, the transmittances of the moth-eye thin film and glass substrate over incidence angles ranging from 0 to 60° were measured. As shown in Fig. 5, the moth-eye film demonstrates considerably better transmittance than the glass substrate. The maximum transmittance of the moth-eye film varies from 96.7 to 95.0% within 0 to 40°, while the maximum transmittance of glass varies from 91.2 to 90.0%. Obviously, the moth-eye thin film shows favorable wide-angle antireflective property. In addition, in contrasts to the thin films consisting of accumulated silica nanoparticles with narrowband antireflection^27,30,31^, the moth-eye thin film shows relative broadband antireflective properties in the wavelength of 400–1500 nm.

**Figure 6:**
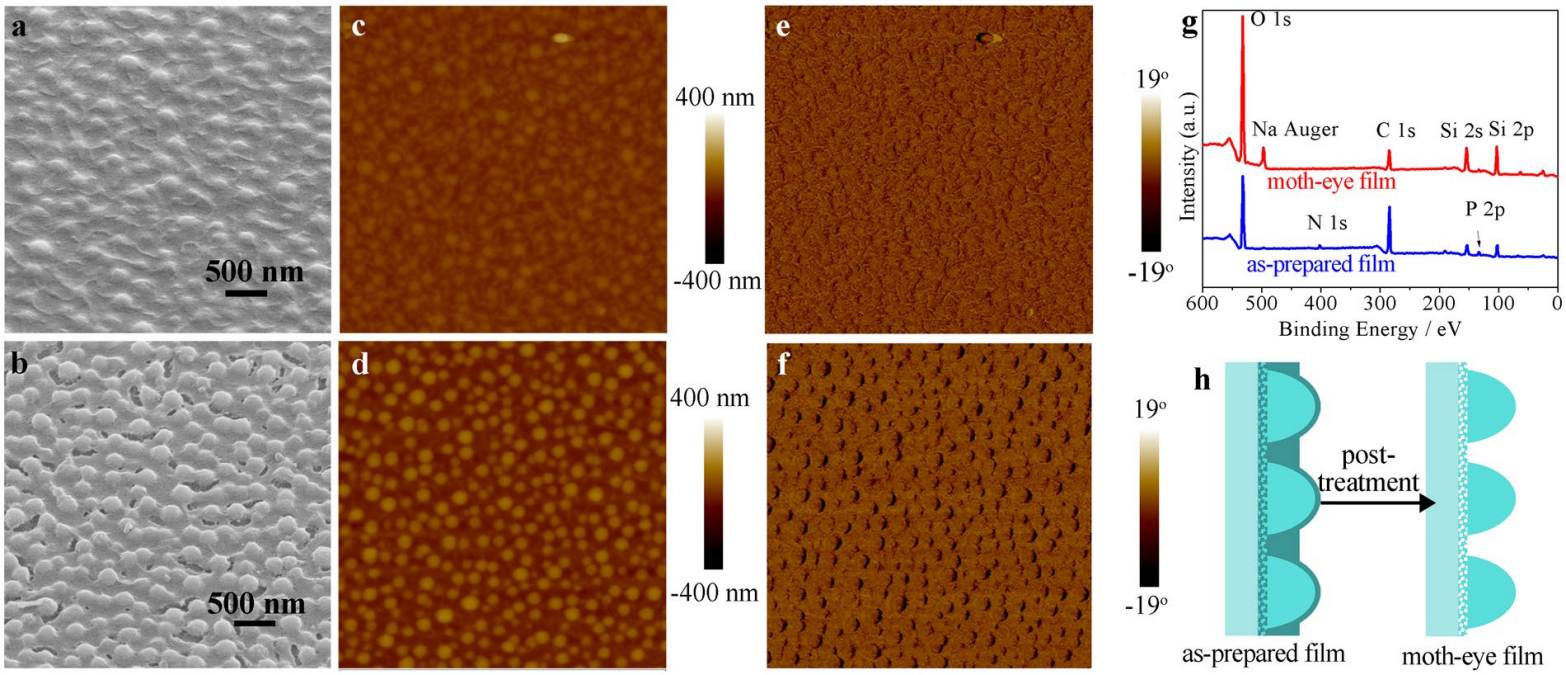
(**a**,**b**) SEM, (**c**,**d**) two-dimensional AFM and (**e**,**f**) AFM phase images (5 × 5 μm^2^) of the as-prepared film and moth-eye film, respectively. (**g**) XPS spectra and (**h**) schematics of the structures of as-prepared film and moth-eye film.

The morphology and chemical composition differences between the as-prepared film and moth-eye film were investigated in order to understand the formation mechanism of the film. As shown in Fig. 6a, the as-deposited film from the sol separated to the phase of homogeneously dispersed nipples and the phase of gaps with the evaporation of solvent. In contrast, the moth-eye film demonstrated a surface structure of clearer nipples (Fig. 6b), indicating that some components in the gaps were apparently removed in the following ultra-sonication washing and calcination process. AFM images (Fig. 6c,d) of the as-prepared film and the final moth-eye film also support this assumption. After washing and calcination, the root-mean-square surface roughness (Rq) of the film increased from 17 nm to 34 nm over a 5 × 5 μm region. Figure 6e and f show the phase images of the as-prepared film and moth-eye film, corresponding to Fig. 6c,d, respectively. The phase images reveal the surface of both films have nearly identical viscoelasticity in the nipples and gaps regions, indicating the nipples and gaps surfaces probably have nearly identical chemical composition. X-ray photoelectron spectroscopy (XPS) was used to reveal the compositional difference between the as-prepared film and the moth-eye film. As shown in Fig. 6g and Table S1, the as-prepared film mainly consists of C, Si, O, N and P elements, while the moth-eye film mainly consists of C, Si and O elements. The N element (from CTAB) peak disappears in the moth-eye film (red line) in comparison with the as-prepared film (blue line), indicating the removal of CTAB. Besides, the content of P element on the moth-eye film also decreased greatly. In detail, the experimental value of P/Si molar ratio of the as-prepared film is 0.23, which is nearly consistent with the theoretical value (0.20). The experimental value of P/Si molar decreased, however, to 0.07 after the post-treatment (washing and calcination). This reveals the removal of the majority of P element. Hence, CTAB and phosphoric acid were probably removed in the post-treatment process. Energy dispersive spectroscopy (EDS) analysis was also performed to investigate the composition difference between the gaps and nipples of the as-prepared film. The data was collected in the nipple and gap regions, respectively (Figure S3). The mole ratios of several major elements to Si (the majority element of the film) are shown in Table S2. Clearly, the molar ratios of C/Si, N/Si, P/Si and O/Si in the gap region are all bigger than those in the nipple region. It is known that the N and C elements are mainly from CTAB and P is from H_3_PO_4_ according to the chemical composition of precursor. Thu, it is reasonable to conclude that the CTAB and phosphorus acid enriched in the gap region and consequently silica enriched in the nipple region. According to the element distribution and the structure of the as-prepared and final moth-eye film, a structure evolution is proposed (Fig. 6h). Initially, as shown in the left part of Fig. 6h, the as-prepared film consists of silica rich region (light green part) and CTAB & phosphorus rich region (dark green part). After post-treatment, components, such as N, P, were removed from the gaps of the as-prepared film, forming a final moth-eye structure (the right part of Fig. 6h).

**Figure 7:**
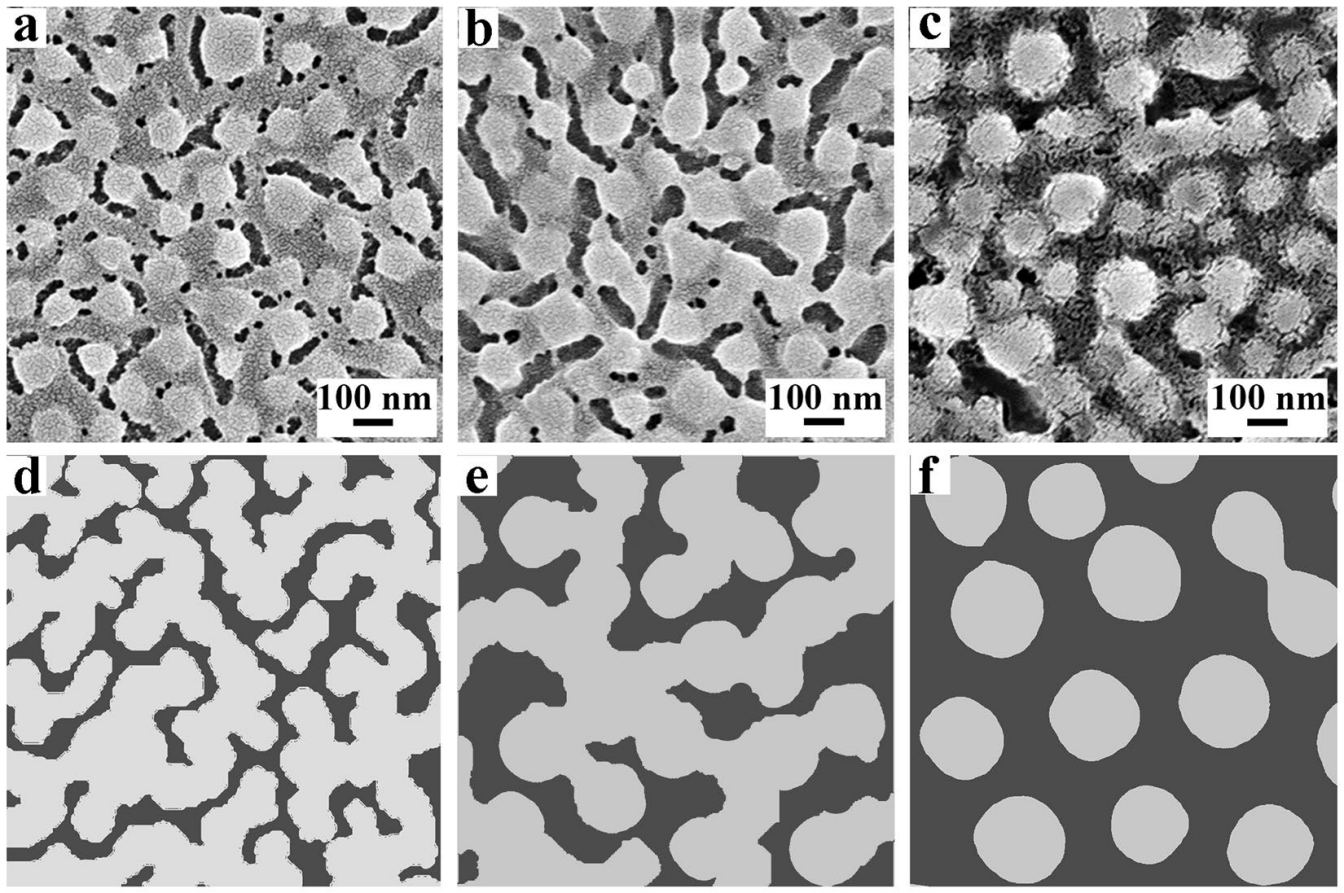
(**a**–**c**) SEM images of con-continuous films with a dip-coating speed of 60, 90, 120 mm/min, respectively. (**d**–**f**) Schematic of microstructural evolution by increasing phase separation time of spinodal decomposition.

The formation mechanism of the moth-eye film was investigated. Dynamic light scattering (DLS, the range of particle size: 2 nm–3 μm) measurements of the sol for preparing coatings showed that there were no particles larger than 2 nm in the pellucid sol. Thus, the nipples (ca. 182 nm) must have formed, probably by phase separation, in the gelation process with the evaporation of solvent when the sol was deposited on substrate. The surface morphology change in phase separation was revealed by freezing the phase separating stages of different time in the process^1^. Whenever the film lose its mobility upon solvent evaporation, the film structure is freezed. Thus, the phase separating stages of varied time could be obtained by regulating the evaporation time of the sol on substrate. Meanwhile, the evaporation time depends on the thickness of the sol on substrate, which could be regulated in turn by the dip-coating speed. Therefore, the different stages of phase separation could be obtained by regulating the dip-coating speed. Namely, the film structures could be freezed in the early stage of phase separation when the dip-coating speed is slow and vice versa. Figure 7a–c show the SEM images of films prepared at a dip-coating speed of 60, 90 and 120 mm/min, respectively. The average size of nipples increases from 90 nm to 115 nm to 139 nm with increase of phase separation time, and the average size of gaps show a similar trend increasing from 32 nm to 45 nm to 64 nm. As the phase separation time increases, the overall morphology of the film changes from unclear small sphere-chains to clear sphere-chains to isolated nipples, revealing both the nipples and gaps evolving with time. When lowering the phase separation speed by regulating the MTMS/TEOS molar ratio of the precursor to 7/3, the surface morphology of the films (Figure S4) revealed even earlier stages in contrast to the films in Fig. 7a–c, demonstrating the phase separation occurs from lines with minor spheres to line-spheres to isolated spheres with evolution time. These time-related phase separation is considered to conform to the spinodal decomposition^1^. Fig. 7d–f show a microstructural evolution by increasing phase separation time of spinodal decomposition under the Cahn–Hilliard equation, which is accompanied by distinctive coarsening and spinodal decompostion^1,11^. The simulated morphological changes nearly conform to the experiment case in Fig. 7a–c, suggesting that the current system may evolve by the spindoal decomposition mechanism.

The phase separation can be further interpreted by the Flory-Huggins solution theory^32^. Methyl-silica oligomers polymerize and form a new silica phase with solvent evaporation, and the new incompatible silica phase then separates from the sol. The Gibbs free energy change of mixing for a binary system can be expressed as:

2 $${\rm{\Delta }}{{\rm{G}}}_{{\rm{M}}}={\rm{\Delta }}{{\rm{H}}}_{{\rm{M}}}-{\rm{T}}{\rm{\Delta }}{{\rm{S}}}_{{\rm{M}}}$$

3 $$\propto \,{\rm{RT}}[{{\rm{\phi }}}_{1}/{{\rm{P}}}_{1}{\mathrm{ln}{\rm{\phi }}}_{1}+{{\rm{\phi }}}_{2}/{{\rm{P}}}_{2}{\mathrm{ln}{\rm{\phi }}}_{2}+{{\rm{\chi }}}_{12}{{\rm{\phi }}}_{1}{{\rm{\phi }}}_{2}],$$

4 $${\rm{\Delta }}{{\rm{H}}}_{{\rm{M}}}\propto {\mathrm{RT}{\rm{\chi }}}_{12}{{\rm{\phi }}}_{1}{{\rm{\phi }}}_{2},{\rm{and}}\,{{\rm{\chi }}}_{12}\propto {({{\rm{\delta }}}_{1}-{{\rm{\delta }}}_{2})}^{2}/{\rm{RT}},$$

5 $$-{\rm{T}}{{\rm{\Delta }}{\rm{S}}}_{{\rm{M}}}\propto {\rm{RT}}[{{\rm{\phi }}}_{1}/{{\rm{P}}}_{1}{\mathrm{ln}{\rm{\phi }}}_{1}+{{\rm{\phi }}}_{2}/{{\rm{P}}}_{2}{\mathrm{ln}{\rm{\phi }}}_{2}]$$

Here, ΔH_M_ and ΔS_M_ denote the changes of enthalpy and entropy at constant temperature and external pressure, φ_i_ and P_i_ (i = 1, 2) denote the volume fraction and the degree of polymerization of each component, χ_12_ is the interaction parameter, which depends on the nature of the components and is estimated from the Hildebrand solubility parameters (δ_i_) of the components. It is noted that the increase of polymerization degree of methyl-silica oligomers would lead to decrease in the absolute value of negative entropic terms and the positive enthalpic term would increases as the polymerization proceeds, both resulting in the increase of ΔG. When △G turns from negative to positive, a driving force of phase separation appears^1^. When d^2^ΔG/dφ^2^ < 0, the system would eventually separate by spinodal decomposition with infinitesimally small fluctuations in composition and density, leading to the phase separation^1,11^.

In summary, we demonstrated *in situ* formation of artificial moth-eye structure from a sol layer deposited on substrate by spontaneous nano-phase separation with solvent evaporation. The prepared thin film had a homogenously distributed nipples array structure with broadband antireflection functionalities. The mechanism of nano-phase separation was unveiled as spinodal decomposition by chemical freezing of the evolution stages and thermodynamic analysis of the system. The current method may provide a new avenue to ready fabrication of patterned nanostructures toward a variety of applications.

## Methods

### Chemicals

Tetraethyl orthosilicate (TEOS, 98%) and methytrimethoxysilane (MTMS, 97%) were purchased from Alfa Aesar. Phosphoric acid (85%), Cetyltrimethyl ammonium bromide (CTAB), hydrochloric acid (38%), and absolute ethanol (99.5%) were obtained from Beihua Fine Chemicals. Ultrapure water with a resistivity higher than 18.2 MΩ•cm was used in all experiments, and was obtained from a three-stage Millipore Mill-Q Plus 185 purification system (Academic).

### Preparation of the sol and films

Hybrid sols were synthesized as follows: MTMS/TEOS/HCl/H_2_O/EtOH with a respective molar ratio of 0.5:0.5:0.004–0.007:3–6:41 were mixed, and then 2–2.5 wt% CTAB and 1.5–4 wt% H_3_PO_4_ were added into the mixture followed by stirring at room temperature for 4 h and aging at room temperature for 60–120 h. Then the sol was deposited on both sides of substrates (ca. 1 mm thick) at room temperature with a relative humidity of 15–20%. Finally, the as-prepared film was solidified at 60 °C, ultrasonically cleaned by water and calcined at 500 °C for 1 h. The smooth thin films were prepared by dip-coating the methyl-silica hybrid sol^29^. The control samples in Fig. 3 were prepared by coating the silica sol on the glass substrates, which consists of silica nanoparticles with a diameter of ca. 40 nm.

### Characterization

Scanning electron microscopy (SEM) observations were carried out on a Hitachi S-4800 field emission scanning electron microscope operated at 5 kV. The thin films were coated with a layer of gold by ion sputtering before SEM observations. Transmission electron microscopy (TEM) observations were carried out on a JEOL JEM-2100F transmission electron microscope at an acceleration voltage of 200 kV. Small pieces of thin film were scratched from substrate, dispersed in ethanol by sonication for 10 min, and added onto a carbon-coated copper grid. The TEM grid with thin film pieces was dried at 60 °C overnight before TEM observation. The morphology and roughness of thin films were characterized by atomic force microscopy (AFM) on an MM8-SYS scanning probe microscope (Bruker AXR). Transmission spectra were recorded using a TU-1901 spectrophotometer (Beijing Purkinje General Instrument Co.). Fourier transform infrared (ATR-FTIR) spectra were recorded on a Varian Excalibur 3100 spectrometer. X-ray photoelectron spectroscopy (XPS) analyses of samples were carried out on an ESCALab220i-XL. Size distribution of the sol was carried out on a Zetasizer Nano ZS90 and particle size analyzer. Transmittance and reflectance were measured using an integrating sphere on a Cary 5000 ultraviolet–visible-near-infrared spectrophotometer with air as ref.^25^. The transmittance of moth-eye thin film and glass substrate over the incidence angles of 0−60° were measured using a rotating sample stage on a Lambda 950 UV/vis spectrometer. The mechanical strength of the samples was measured by the sand abrasion test. In detail, sand grains (40 g, 100 to 300 μm in size) impinged the coating surface from a height of 1 m within 40–45 s. The coating tilted 45° with the vertical sand flow.

## Electronic supplementary material

Supplementary Information
