# Supplementary material for: In situ formation of artificial moth-eye structure by spontaneous nano-phase separation
Source: Sci Rep. 2018 Jan 18;8:1082. doi: 10.1038/s41598-018-19414-x (PMC5773675; doi:10.1038/s41598-018-19414-x)
Supplement: Supplementary file 1 — Supplementary Information [file 41598_2018_19414_MOESM1_ESM.doc]

Supporting Information

**Title** In situ formation of artificial moth-eye structure by spontaneous nano-phase separation

*Tong Li,^a,b^ Junhui He,^a,*^ Yue Zhang,^a^ Lin Yao,^a^ Tingting Ren^a,b^ and Binbin Jin^a,b^*

a Functional Nanomaterials Laboratory, Center for Micro/Nanomaterials and Technology, and Key Laboratory of Photochemical Conversion and Optoelectronic Materials, Technical Institute of Physics and Chemistry, Chinese Academy of Sciences, Zhongguancundonglu 29, Haidianqu, Beijing 100190, China.

b University of Chinese Academy of Sciences, Beijing 100049, China.

* Corresponding author. E-mail address: jhhe@mail.ipc.ac.cn.


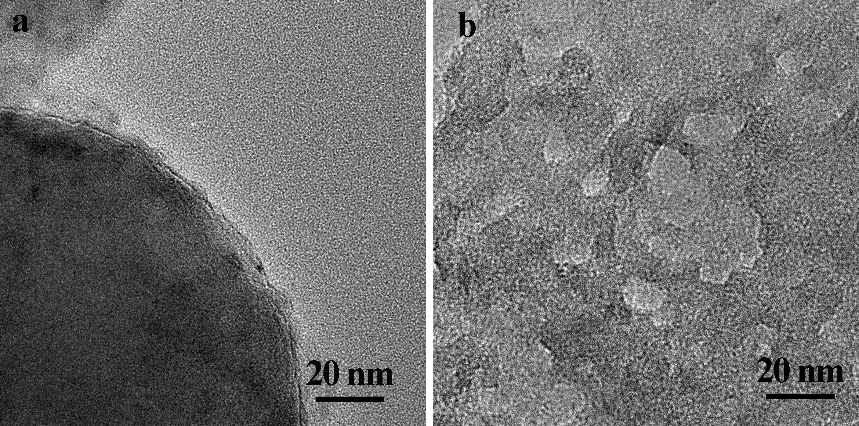


Figure S1 High magnification TEM images of the nipple (a) and gap (b) regions. Black arrows and red arrow point to nanopores and a larger pore, respectively.

**Table S1** The P/Si and N/Si molar ratios of the as-prepared and moth-eye

films by XPS analyses in contrast to the theoretical values.

|  | **Theoretical value** | **As-prepared film** | **Moth-eye film** |
| --- | --- | --- | --- |
| n_P/Si_ | 0.2 | 0.23 | 0.07 |
| n_N/Si_ | 0.14 | 0.16 | 0 |


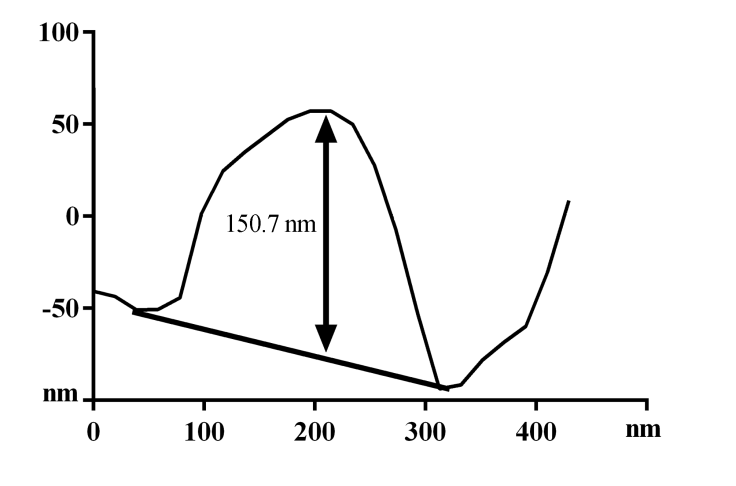


**Figure S2** The height profile of a nipple as an example.


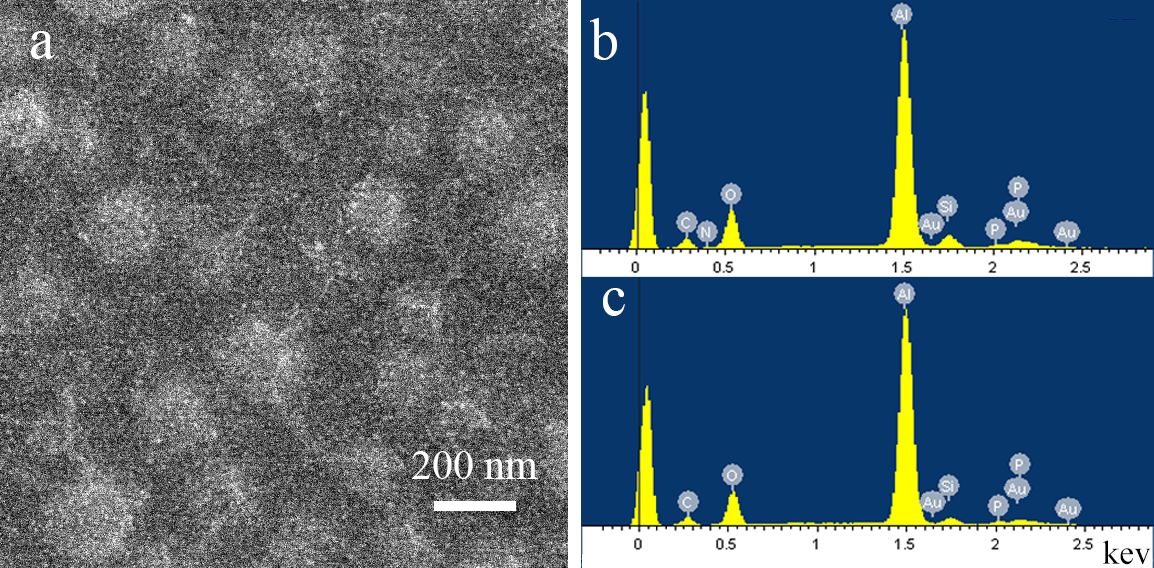


**Figure S3** (a) SEM image and (b,c) EDS analyses of nipples and gaps of the as-prepared film (the arrows point to the positions analyzed).

**Table S2** The P/Si, N/Si, C/Si and O/Si molar ratios of

the as-prepared film by EDS analysis

|  | nipples | gaps |
| --- | --- | --- |
| **n_O/Si_** | 15.4 | 25.5 |
| **n_P/Si_** | 0.22 | 0.36 |
| **n_C/Si_** | 14.7 | 21.8 |
| **n_N/Si_** | 1.81 | 3.11 |


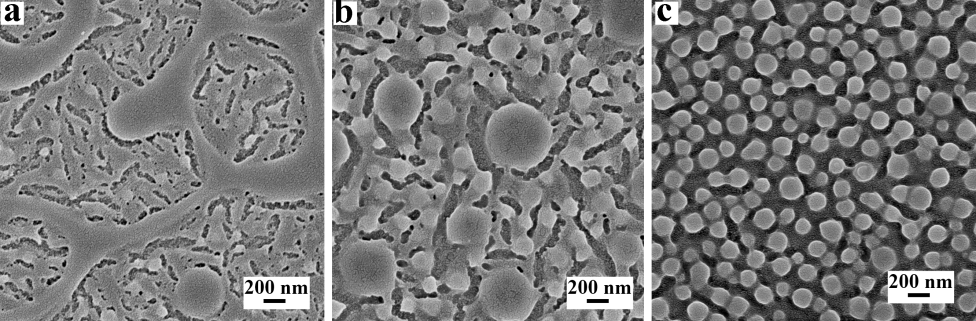


**Figure S4** (a-c) SEM images of films prepared by a precursor of a MTMS/TEOS molar ratio of 7/3 using a dip-coating speed of 60, 90, 120 mm/min, respectively.
